# Quantum-State Controlled Reaction Channels in Chemi-ionization Processes: Radiative (Optical–Physical) and Exchange (Oxidative–Chemical) Mechanisms

**DOI:** 10.1021/acs.accounts.0c00371

**Published:** 2020-09-15

**Authors:** Stefano Falcinelli, James M. Farrar, Franco Vecchiocattivi, Fernando Pirani

**Affiliations:** † Dipartimento di Ingegneria Civile ed Ambientale, Università di Perugia, 06125 Perugia, Italy; ‡ Department of Chemistry, University of Rochester, Rochester, New York 14627, United States; § Dipartimento di Chimica, Biologia e Biotecnologie, Università di Perugia, 06123 Perugia, Italy; ∥ Istituto di Scienze e Tecnologie Chimiche “G. Natta” CNR-SCITEC, 06123 Perugia, Italy

## Abstract

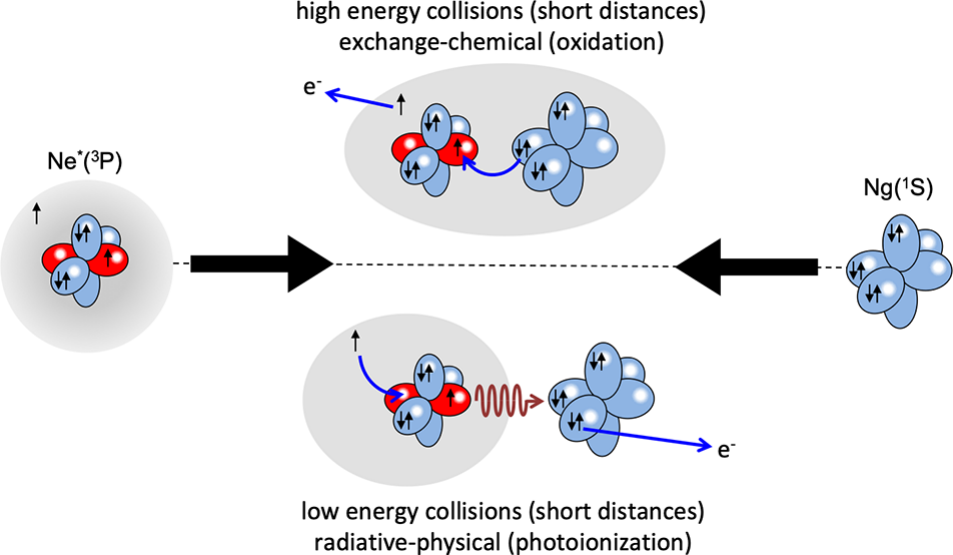

Most chemical processes are triggered by electron or charge transfer phenomena (CT). An important class of processes involving CT are chemi-ionization reactions. Such processes are very common in nature, involving neutral species in ground or excited electronic states with sufficient energy (X*) to yield ionic products, and are considered as the primary initial step in flames. They are characterized by pronounced electronic rearrangements that take place within the collisional complex (X···M)* formed by approaching reagents, as shown by the following scheme, where M is an atomic or molecular target: X* + M → (X···M)* → [(X^+^···M) ↔ (X···M^+^)]^*e*−^ (X···M)^+^ + e^–^ → final ions.

Despite their important role in fundamental and applied research, combustion, plasmas, and astrochemistry, a unifying description of these basic processes is still lacking. This Account describes a new general theoretical methodology that demonstrates, for the first time, that chemi-ionization reactions are prototypes of gas phase oxidation processes occurring via two different microscopic mechanisms whose relative importance varies with collision energy, *E*_c_, and separation distance, *R*. These mechanisms are illustrated for simple collisions involving Ne*(^3^P_2,0_) and noble gases (Ng). In thermal and hyperthermal collisions probing interactions at intermediate and short *R*, the transition state [(Ne···Ng)^+^]^*e*−^ is a molecular species described as a molecular ion core with an orbiting Rydberg electron in which the neon reagent behaves as a halogen atom (i.e., F) with high electron affinity promoting chemical oxidation. Conversely, subthermal collisions favor a different reaction mechanism: Ng chemi-ionization proceeds through another transition state [Ne*······Ng], a weakly bound diatomic-lengthened complex where Ne* reagent, behaving as a Na atom, loses its metastability and stimulates an electron ejection from M by a concerted emission–absorption of a “virtual” photon. This is a physical radiative mechanism promoting an effective photoionization. In the thermal regime of *E*_c_, there is a competition between these two mechanisms. The proposed method overcomes previous approaches for the following reasons: (1) it is consistent with all assumptions invoked in previous theoretical descriptions dating back to 1970; (2) it provides a simple and general description able to reproduce the main experimental results from our and other laboratories during last 40 years; (3) it demonstrates that the two “exchange” and “radiative” mechanisms are simultaneously present with relative weights that change with *E*_c_ (this viewpoint highlights the fact that the “canonical” chemical oxidation process, dominant at high *E*_c_, changes its nature in the subthermal regime to a direct photoionization process; therefore, it clarifies differences between the cold chemistry of terrestrial and interstellar environments and the energetic one of combustion and flames); (4) the proposed method explicitly accounts for the influence of the degree of valence orbital alignment on the selective role of each reaction channel as a function of *E*_c_ and also permits a description of the collision complex, a rotating adduct, in terms of different Hund’s cases of angular momentum couplings that are specific for each reaction channel; (5) finally, the method can be extended to reaction mechanisms of redox, acid–base, and other important condensed phase reactions.

## Key References

- FalcinelliS.; VecchiocattiviF.; PiraniF.Adiabatic and Nonadiabatic Effects in the Transition States of State to State Autoionization Processes. Phys. Rev. Lett.2018, 121, 1634033038766910.1103/PhysRevLett.121.163403.^1^*New insights are provided on the electronic adiabatic and nonadiabatic effects in the stereodynamics of state to state atomic and molecular collisions, controlling relevant properties of the transition state of chemi-ionization reactions*.
- FalcinelliS.; PiraniF.; CandoriP.; BrunettiB. G.; FarrarJ. M.; VecchiocattiviF.A new insight of stereo-dynamics of Penning ionization reactions. Front. Chem.2019, 7, 4453127592610.3389/fchem.2019.00445PMC6591474.^2^*Recent developments in the experimental study of chemi-ionization reactions are presented to cast light on basic aspects of the stereodynamics of the microscopic mechanisms involved*.
- FalcinelliS.; VecchiocattiviF.; PiraniF.General treatment for stereo-dynamics of state-to-state chemi-ionization reactions. Commun. Chem.2020, 3, 6410.1038/s42004-020-0312-3PMC981470036703400.^3^A theoretical approach able to formulate the optical potential for Ne*(^3^P_2,0_) noble gas atom chemi-ionizations as prototype oxidation processes and to evaluate the state-to-state reaction probability is proposed.

## Introduction

Anisotropic intermolecular forces, associated with alignment and orientation effects produced by atomic and molecular polarization, modulate the fate of molecular collisions. A knowledge of these phenomena is relevant to control the stereodynamics of elementary processes occurring in the gas phase and at the gas–surface interface,^4−18^ but a general theoretical and computational foundation is still lacking.

This Account focuses on the role of valence atomic orbital alignment in determining the selectivity of electronic rearrangements that affect the stereodynamics of gas-phase chemi-ionization reactions (Penning ionization phenomena).^19−22^ Our study provides complementary information to the nuclear stereodynamics deeply investigated in seminal works.^23−25^ Indeed, present atom–atom reactions are directly triggered by the electronic rearrangements and indirectly affected by nuclear motions: possible electronic–nuclear couplings emerge as Coriolis effects.

Chemi-ionization reactions occur in collisions of open shell species, electronically excited in energetic metastable states, with neutral partners, giving rise to spontaneous ejection of electrons and subsequent ion formation. The reactions proceed without a barrier and are described by an anisotropic optical potential, *W*, defined in eq 1 as a combination of a real (*V*_t_) and an imaginary (Γ) part that control, respectively, entrance–exit channel trajectories and disappearance probability of neutral reactants by ionization.^19−22,26,27^
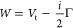
1The strength of both the real and imaginary components varies with the center-of-mass separation and relative orientation of interacting partners. The imaginary component Γ mediates the passage from neutral reactants to ionic products through an electronic rearrangement within the reaction transition state (TS).

Chemi-ionization processes studied under electronically state-selected conditions are important for catalysis, plasmas, photodynamics, and interstellar and low-temperature chemistry and play an important role in applied research topics such as soft ionization in mass spectrometry.^28−31^ Such reactions are the primary step in flames,^32,33^ classified here as prototypes of strongly exothermic elementary oxidation processes, for which the details of the stereodynamics are provided by Penning ionization energy spectra (PIES) of spontaneously emitted electrons and by total and partial ionization cross sections.^12,21^ These experimental observables are very sensitive probes that highlight the crucial features of TSs such as geometry and orbital energetics.

This Account focuses on reactions of metastable Ne*, with a valence electron excited to a 3s orbital. Its open-shell ionic core Ne^+^ exhibits the same electronic configuration, 2p^5^, of the high electron affinity fluorine atom, with ^2^P_3/2,1/2_ fine structure levels. When Ne* approaches an atomic or molecular target M with sufficient collision energy (*E*_c_), it forms an interacting complex within which a spontaneous electron jump from one of the HOMOs (highest occupied molecular orbitals) of M to the open shell ionic core of Ne* can occur, releasing enough energy to eject the 3s electron with a defined kinetic energy. Therefore, measured PIES^34,35^ provide direct information on electronic rearrangements occurring inside the TS.^36^ Moreover, the ionization probability and PIES are strongly dependent on symmetry, energy, and relative spatial orientation of the atomic or molecular orbitals involved in the electron exchange.

A number of laboratories including our own fully highlighted the reaction dependence on the orbital orientation of various molecular systems.^37−40^ However, in the case of the anisotropic Ne* reagent, an important open question concerns the selective role of the half-filled 2p atomic orbital within the collision complex the alignment of which affects the TS structure. To emphasize basic aspects of the stereodynamics promoted by selective electronic rearrangements, we have focused on prototype atom–atom reactions between Ne* and the heavier noble gases (Ng = Ar, Kr, Xe). The limited internal degrees of freedom of Ng, the absence of fragmentation in Ng^+^ product, and the availability of detailed experimental findings such as cross sections, branching ratios (BRs), and PIES facilitated the investigation.

Ours is an innovative theoretical approach based on identification and modeling of the basic components of the interaction. Their formulation uses fundamental physical properties as scaling parameters (polarizability, ionization potential, electronic affinity, spin–orbit (SO) splitting) of the participating collisional partners, providing a computational method based on simple-operating interdependent relationships.

Our study on Ne*–Kr^1,2^ serves as a paradigm for emphasizing similarities and differences in the reaction stereodynamics of the complete Ne*–Ng family.

The computational method, which provides an integrated picture of the stereodynamics of this series of chemi-ionization reactions, is based on two *important markers*, *C*_*x*_ and *C*_*y*,_ which quantify the Σ character degree in excited and lowest electronic states, respectively, of the molecular ion (Ne···Ng)^+^ coupled by CT. Such markers, identifying how the molecular symmetry degree of the state-selected collision complexes (which evolve in the TS ones at the turning point region) changes with the interatomic distance *R*, represent how quantum levels of reagents and products couple during each collision event. They describe how the SO levels of reagents and products are perturbed at large *R* and destroyed at shorter *R* by increasing strength and anisotropy of the electric field associated with the interaction. Only strong electric fields decouple the electronic orbital angular momentum from the spin and effectively align valence orbitals along **R**, promoting the formation of real molecular states.

Therefore, the markers map all reaction dynamics changes as a function of *E*_c_ and concomitant changes in the ranges of *R* probed.

This approach emphasizes intriguing microscopic aspects of the processes that had not been previously considered:

(i) Entrance and exit channels belong to a manifold of states of the same system, properly coupled by the configuration interaction. Their characterization provides the correct sequence in energy of quantum levels accessible, including also those of the TS, and their dependence on *R*; the real and imaginary parts of the optical potential are interdependent, being related to *adiabatic* and *nonadiabatic* effects, respectively, arising from electronic rearrangements occurring within the collision complex.
(ii) The microscopic mechanisms triggered by the selectivity of interaction components have a marked *E*_c_ dependence:
  *Subthermal conditions* promote reactions classified as photoionization processes, where only long-range noncovalent interactions (induction, dispersion, and polarization) are effective. They determine the formation of weakly bound diatomic adducts [Ne*······Ng] (the TS in this case) where Ne* behaves as a sodium atom perturbed by the Ng presence: this breaks the validity of the optical selection rules, allowing ionization to occur by a concerted emission–absorption of a “virtual” photon.^3,41^
  *Hyperthermal conditions* favor processes that evolve as chemical oxidation reactions, where the TS is a molecular complex of which the accessible levels are represented by proper molecular quantum numbers. In this case, the collision complex [Ne*······Ng] formed at large *R* does not ionize and evolves toward shorter *R* where the Ne* polarization makes the stronger ion–dipole interaction effective, trapping the reactants via the formation of [(Ne···Ng)^+^]^*e*−^ TS:
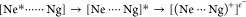
2Here, the behavior of neon is dominated by its ionic core (behaving as a fluorine atom) inducing the oxidation of the Ng via an electron transfer. Under thermal conditions, the two types of reactions occur simultaneously, and their relative role varies with *E*_c_ depending on both reaction channel and Ng characteristics.

## Computational Methodology

The proposed methodology exploits the following steps suggested by our recent research.^1−3^

### Optical Potential Formulation

The real part, *V*_t_, of eq 1 assumes that each entrance channel is determined by the weighted sum of two limiting representations.^1−3^ At large *R*, the system exhibits a substantial isotropic behavior, typical of an alkaline atom interacting with Ng and promoting a photoionization (physical) process. At intermediate and short *R*, the anisotropy of the ionic core of Ne* emerges, behaving as a F atom, which promotes an oxidation reaction. The interaction in the entrance channels must take into account the anisotropic contributions from the open shell “P” nature of Ne*,^1−3,21,22,41−47^ whereas the exit channels are affected by the P nature of the Ng^+^ product.

The investigation of the interaction of open P shell atoms or ions with a closed shell ^1^S_0_ species^47,48^ suggests a *V*_t_ representation defined in terms of proper quantum numbers that accounts for the relative alignment or orientation of reagents and products within the interatomic electric field, which is the proper quantization axis of the system. The resultant interactions provide effective *adiabatic* potentials that include *V*_Σ_ and *V*_Π_ contributions mixed by SO effects. The Σ and Π molecular states are defined by the electronic quantum number Λ = 0 and Λ = 1, where Λ describes the absolute projection of the orbital angular momentum decoupled by the spin along **R**. For a full description of present anisotropic interactions, it is sufficient to use^42^ a weighted sum of *V*_0_ and *V*_2_ Legendre-expansion radial coefficients:^47,48^
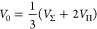
3
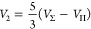
4*V*_0_ represents the isotropic component, with all anisotropic contributions included in the *V*_2_ term. The latter, accounting for the quantized spatial orientation of valence orbitals of the open shell species within the interacting complex, controls the sequence in the manifold of *adiabatic* potential energy curves^47,48^ (PECs) associated with all quantum states accessible, including their stabilities and anisotropies. Accordingly, for all channels, the effective PECs have been formulated^3^ and indicated as *V*_|*J*,Ω⟩_ (*J* is the total (orbital + spin) electronic angular momentum quantum number, while Ω is the absolute projection of **J** along **R**).

While the isotropic *V*_0_ term is a noncovalent interaction component, the anisotropic *V*_2_ originates primarily from “chemical” contributions. In entrance channels, *V*_0_ accounts for the gradual passage of the system, as *R* decreases, from neutral–neutral [Ne*······Ng] to ion–neutral [(Ne···Ng)^+^]^*e*−^, that is, a molecular ion core surrounded by a Rydberg electron (eq 2).^1−3^ In exit channels, it is determined by an isotropic Ne···Ng^+^ ion–neutral interaction. In both cases, *V*_0_ depends on size repulsion, polarization, and dispersion/induction attraction contributions. In contrast, *V*_2_ identifies the anisotropic configuration interaction (CI) between entrance and exit channels differing for one electron exchange. *V*_2_ is represented by an exponential decreasing function of *R*,^3,47,48^ reflecting the “canonical” dependence of the integral overlap between atomic orbitals exchanging the electron. For entrance and exit channels, the modulus of the exponential function must be the same, while its sign is negative for the exit channel and positive for the entrance channel. The different signs relate to *bonding* and *antibonding* effects by charge or electron transfer (CT) that arise from the CI between entrance and exit channels of the same symmetry.^1−3,47,48^ CI makes entrance and exit channels of each system as belonging to the same correlated manifold of states. The formulation of the potential functions is summarized in Supporting Information (SI).

**Figure 1:**
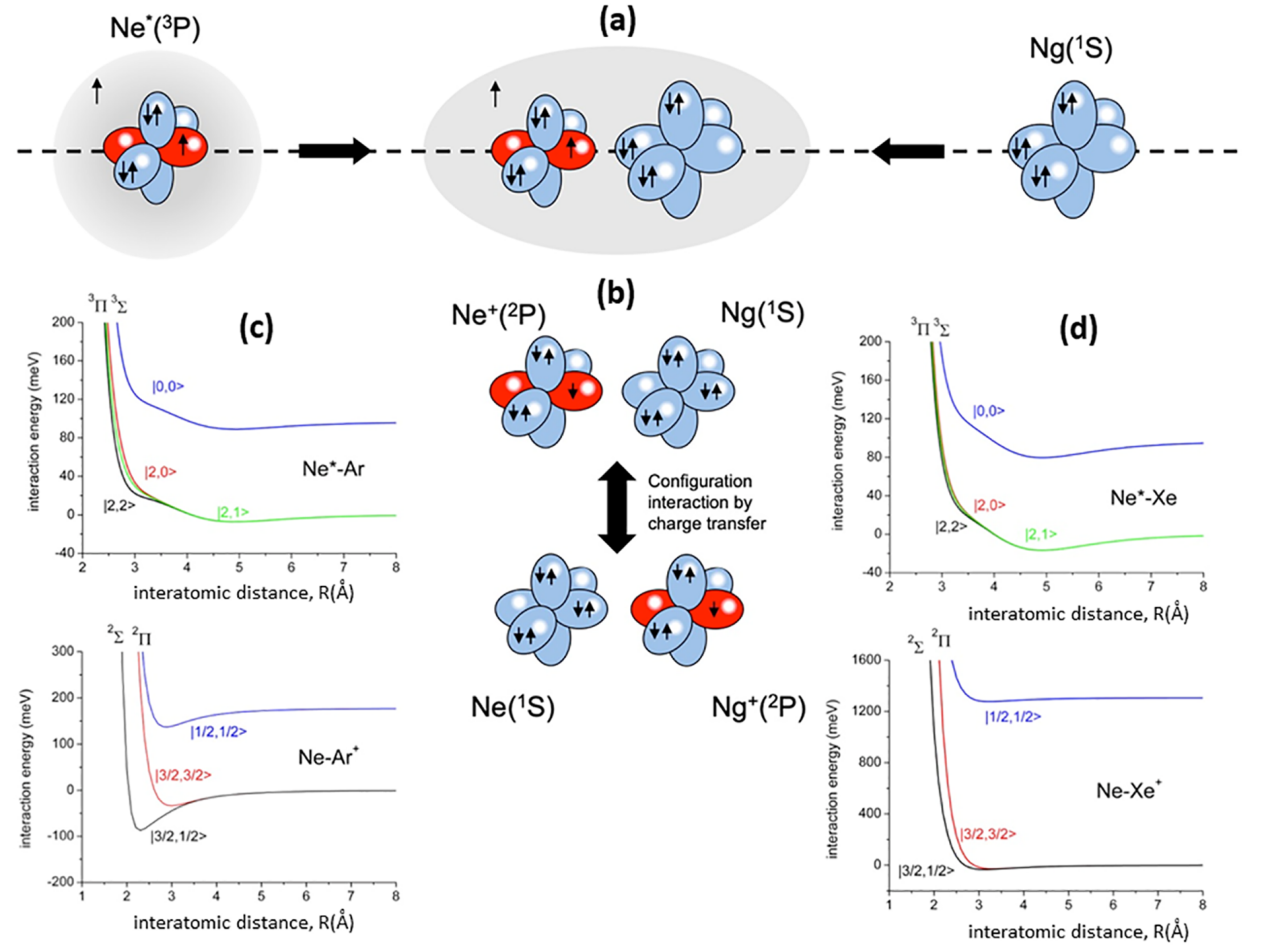
(a) Electronic features of reagents and collision complex. (b) CI between states of entrance and exit channels differing for one electron exchange, defining CT contributions for Σ states. Real part of *W* for Ne*–Ar (c) and Ne*–Xe (d) represented by adiabatic PECs.

For entrance and exit channels of the same system, this approach leads to a different correlation between atomic states, representative of the behavior at long *R*, where |*V*_2_| ≪ SO energy splitting, and molecular states emerging at short *R*, where |*V*_2_| ≫ SO splitting^48^ (Figure 1 and Figure S2). The Σ and Π molecular character degree associated with each *V*_|*J*,Ω⟩_ curve at all *R* values can be evaluated by relations (see SI) that depend on the ratio between *V*_2_ strength and SO splitting and agree with the following asymptotic conditions (Figure 1): at short *R*, all PECs must represent states having pure Σ or Π molecular character, while at large *R*, where the SO coupling is dominant, a mixing of molecular characters occurs.

As previously noted, the adoption of *C*_*x*_ and *C*_*y*_ coefficients quantifies the Σ character degree in entrance and exit channels, respectively: emphasizing all basic electronic rearrangements within the collision complexes, they represent important markers of the reaction dynamics modulation under different conditions. Their characterization is important to provide suitable correlation between atomic and molecular states and to obtain a simple-operating formulation of the imaginary part Γ of the optical potential internally consistent with that of the real part *V*_t_. Indeed, the relative role of Σ and Π molecular character in entrance and exit channels must be properly taken into account to define their couplings and state-to-state Γ components.

### Adiabatic and Nonadiabatic Effects in the Open-Shell Atom Phenomenology

The electronic structure of the Ne* reagent is depicted in Figure 1, where the “floppy” cloud of the outer 3s electron and the nature of the open shell of the ionic core are emphasized. These features determine basic characteristics of the collision complex with the Ng and of the reaction TS. Electronic rearrangements driving the reaction arise from polarization of the 3s electron cloud, CT, and modifications of angular momentum couplings of valence electrons within the collision complex. Such rearrangements are accompanied by adiabatic and nonadiabatic effects, which play a crucial role in the collision dynamics.

Anisotropic adiabatic effects arise from the strength and selectivity of CI within the collision complex, promoted by CT, that couple entrance and exit channels of the same symmetry. Such effects, determining the anisotropy of *V*_t_, account for the adiabatic conversion of atomic states, represented by |*J*,Ω⟩ quantum numbers, into molecular states of Σ and Π symmetry. While the atomic states are representative of reagents and products at large and intermediate *R*, the molecular states of the interacting system emerge at chemical bonding length scales. The resulting PECs for Ne*–Ar and Ne*–Xe systems are plotted in Figure 1 (for Ne*–Kr see ref (3)). The figure depicts also CI and CT for Σ states: the corresponding components for Π states are much smaller^1,2^ because of the reduced overlap integral between atomic half-filled orbitals exchanging the electron, aligned orthogonal to **R**. Sequence and stability of levels, obtained by general guidelines,^48^ are consistent with results of Dehmer^49^ and the natural bond order method.^50^

**Figure 2:**
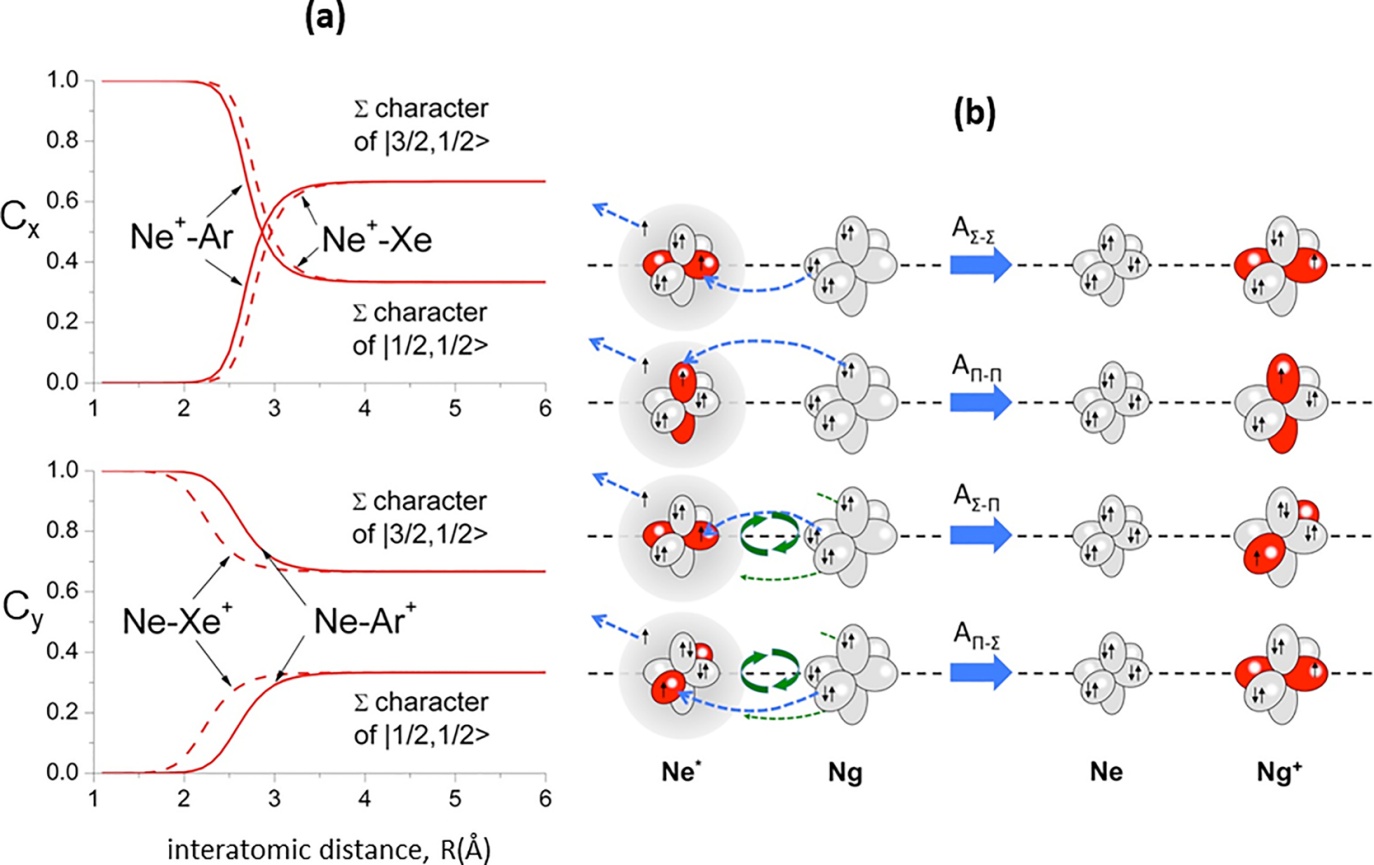
(a) Vertical axes give values of the Σ character in entrance (*C*_*x*_) and exit (*C*_*y*_) channels as a function of *R*. Π character is defined as complement to 1 of the Σ one. All states accessible to the system are indicated by |*J*,Ω⟩ quantum numbers. The |3/2,3/2⟩ states are not included since they exhibit a pure Π character at all *R*. Dashed and full lines refer to Ne–Xe and Ne–Ar systems, respectively. The larger interaction anisotropy of the Ne^+^–Xe system makes the variation of C_*x*_ more prominent with respect to Ne^+^–Ar, while the larger SO coupling of Xe^+^ with respect to Ar^+^ hinders the passage to the molecular state causing less variation of C_*y*_. (b) Cartoon representing the main features of Σ–Σ, Π–Π, Σ–Π, and Π–Σ couplings promoted by *nonadiabatic effects* operative during the collisions.

The components of the imaginary part of the optical potential depend on the strength and radial dependence of nonadiabatic effects. They arise from polarization, selective CI, changes in electronic angular momentum couplings, and SO and Coriolis contributions^1^ as determined in the recent analysis of the Ne*–Kr case.^47^ This procedure exploits the characterization of *C*_*x*_ and *C*_*y*_ discussed above. Figure 2a indicates that at large *R* such coefficients maintain their asymptotic values and the system is not reactive. As *R* decreases, the system is initially affected by weak noncovalent components of the interaction: the *C*_*x*_ and *C*_*y*_ coefficients are slowly varying, with a perturbation of Ne* sufficient to promote within [Ne*······Ng] emission–absorption of a “virtual” photon^41^ initiating a photoionization mechanism. Conversely, at short *R* stronger “chemical” interaction components promote pronounced changes in angular momentum couplings with the passage from atomic to molecular states: the TS, [(Ne···Ng)^+^]^*e*−^, becomes a molecular ion surrounded by a Rydberg electron. In this region, the reactions become true chemical (oxidation) processes. Therefore, *C*_*x*_ and *C*_*y*_, the important markers controlling the relative role of reaction mechanisms accounting for the variation with *R* of the Σ character of the state-selected TS, have been obtained with a procedure detailed in SI and are shown in Figure 2a for Ne*–Ar and Ne*–Xe systems. The *R* interval where they show the fastest variations corresponds to the region where the interaction anisotropy becomes comparable to the SO coupling and an emerging transition from atomic and to molecular states occurs. The behavior of *V*_|2,2⟩_ and *V*_|3/2,3/2⟩_ curves, effective in the entrance and exit channels, respectively, is not discussed in detail since they show at all distances pure Π character.

### Chemi-ionization Reaction Mechanisms

**Figure 3:**
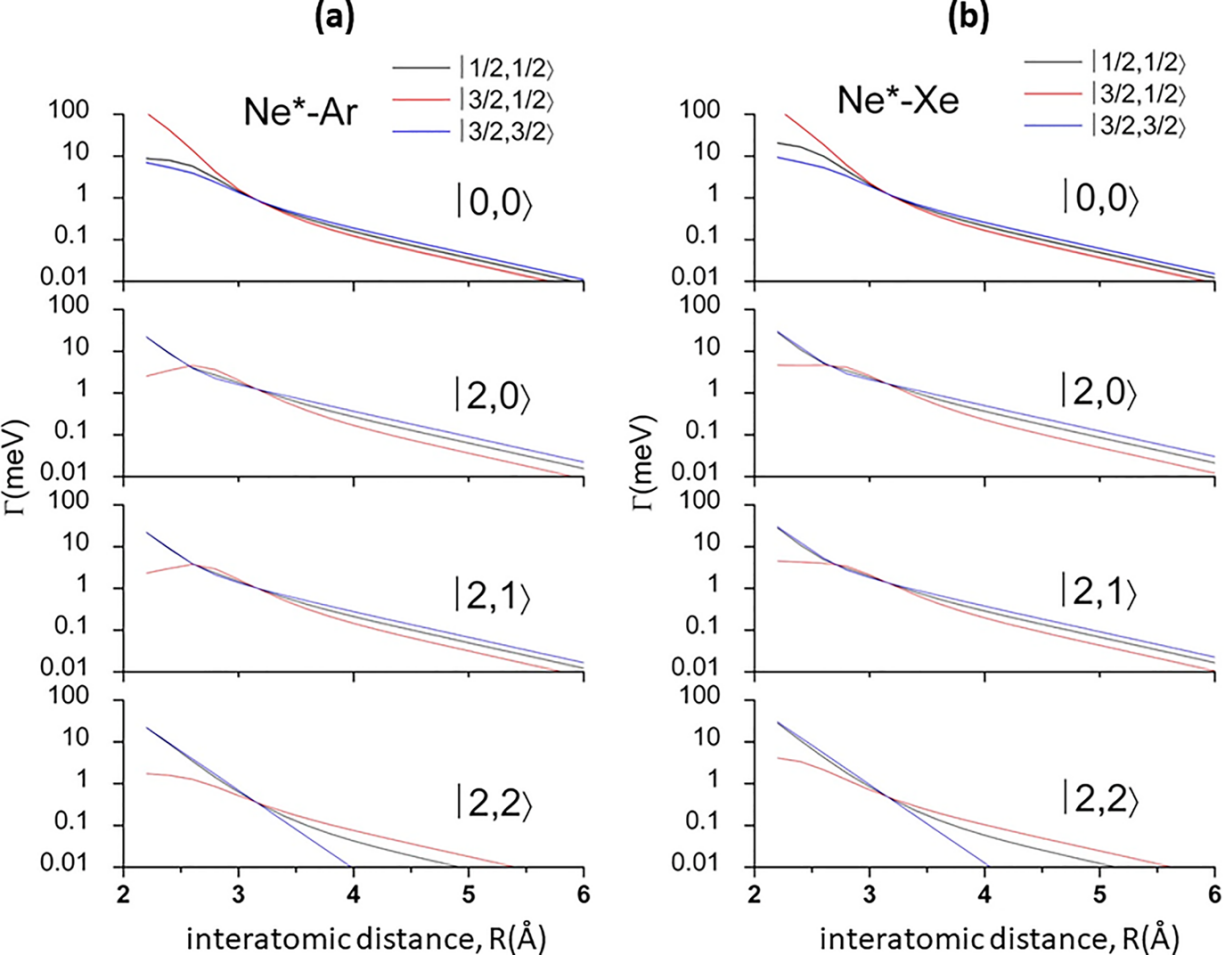
State-to-state Γ components defined in terms of |*J*,Ω⟩ quantum numbers of Ne*(^3^P_J_) reagent and of Ar^+^(^2^P_J_) (a) and Xe^+^(^2^P_J_) (b) products.

The nature of nonadiabatic effects, coupling reagents, and products during collision events suggests that chemi-ionization reactions occur through two complementary microscopic mechanisms, illustrated in the right panel of Figure 3. They are classified as:^1^

(i) *direct mechanism* (driven by “chemical” forces), ΔΛ = 0, with coupling terms called *A*_Σ–Σ_ and *A*_Π–Π_ on the basis of the molecular character (Σ or Π) of initial and final states;
(ii) *indirect mechanism* (controlled by of “physical” forces), ΔΛ= ±1, promoted by electronic polarization, SO, and Coriolis effects and stimulated by mixing between initial and final states of different symmetry, whose coupling terms are defined as *A*_Σ–Π_ and *A*_Π–Σ_.

The two mechanisms show different radial dependence,^1−3^ and therefore their relative roles vary with *E*_c_. The direct mechanism dominates at shorter distances, accessible in higher *E*_c_, and arises from the chemical oxidation of the Ng controlled by the [(Ne···Ng)^+^]^*e*−^ TS, where the Ne* ionic core behaves like a fluorine atom, while the indirect one emerges at lower relative energies when the collision probes larger distances and evolves along PECs dominated by the behavior of Ne* as a sodium atom. The indirect mechanism includes radiative contributions as proposed in pioneering works.^27,41,42^

All coupling terms *A*_Λ__–Λ′_ are represented by exponential functions^1−3^ given in SI. The couplings *A*_Σ–Σ_ and *A*_Π–Π_ exhibit a pronounced radial dependence since they, as the *V*_2_ component, relate to the variation of valence orbital overlap integrals with *R*. However, arising from noncovalent interaction and Coriolis contributions, *A*_Σ–Π_ and *A*_Π–Σ_ show a less pronounced radial dependence.^1−3^ Coupling with the continuous states of the emitted electron,^41,42^ which slowly varies with *R*, is accounted in the pre-exponential factor of *A*_Λ__–Λ′_.

Strength and radial dependence of state-to-state Γ terms have been defined exploiting *A*_Σ–Σ_, *A*_Π–Π_, *A*_Σ–Π_, and *A*_Π–Σ_ terms. Similar to Ne*–Kr,^3^ for the present systems strength and radial dependence of *A*_Σ–Σ_ and *A*_Π–Π_ have been estimated from strength and radial dependence of CI, which couples and mixes states of the same symmetry to which partial or full molecular character can be properly assigned.

Therefore, considering the correlation diagram between atomic and molecular states^3^ reported in SI, we obtained explicit relations for state-to-state Γ_|*J*Ω→*J*′Ω′⟩_ terms of the optical potential (see SI), represented as weighted averages of *A*_Λ__–Λ′_ couplings, where relative weights in each channel are given as combination of *C*_*x*_ and *C*_*y*_.

Such state-to-state Γ_|*J*Ω→*J*′Ω′⟩_ components, reported in Figure 3, exhibit similar behavior for both systems, although for Ne*–Xe they are stronger suggesting the occurrence of more efficient chemi-ionization processes. For the direct mechanism, this is due to the effect of larger atomic overlap of Xe with respect to Ar, while for the indirect mechanism, the larger electronic cloud of Xe causes a higher perturbation on the external electronic configuration of Ne* with subsequently more probable violation of the selection rules favoring its radiative decay.

## Predictions and Experimental Results

**Figure 4:**
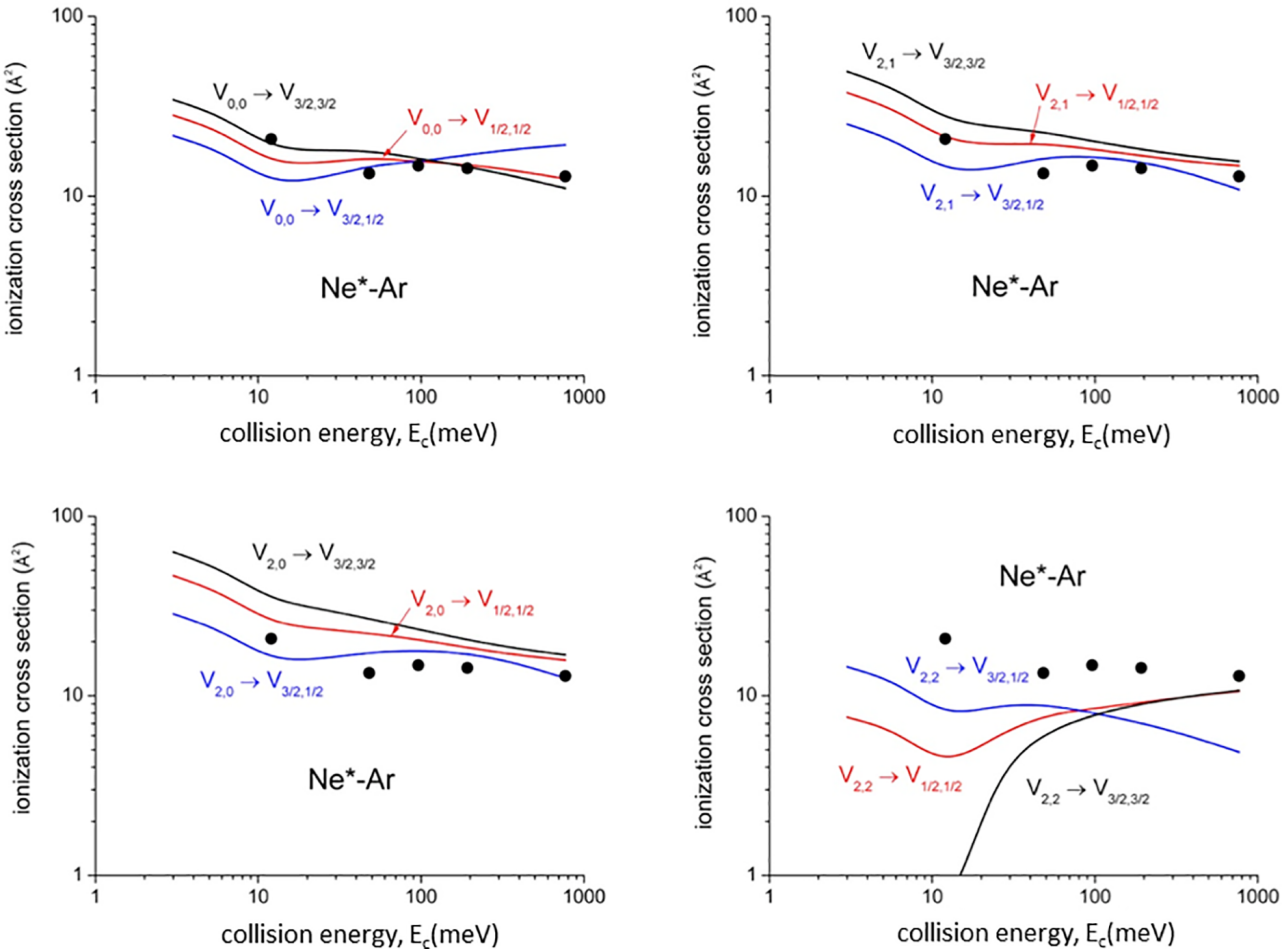
State-to-state total ionization cross section for Ne*–Ar as a function of *E*_c_. The comparison with early experimental results (black points, data from ref (42)) refers to state averaged conditions and emphasizes differences with respect to state-to-state results, while their statistical average is consistent with the experimental determination.

**Figure 5:**
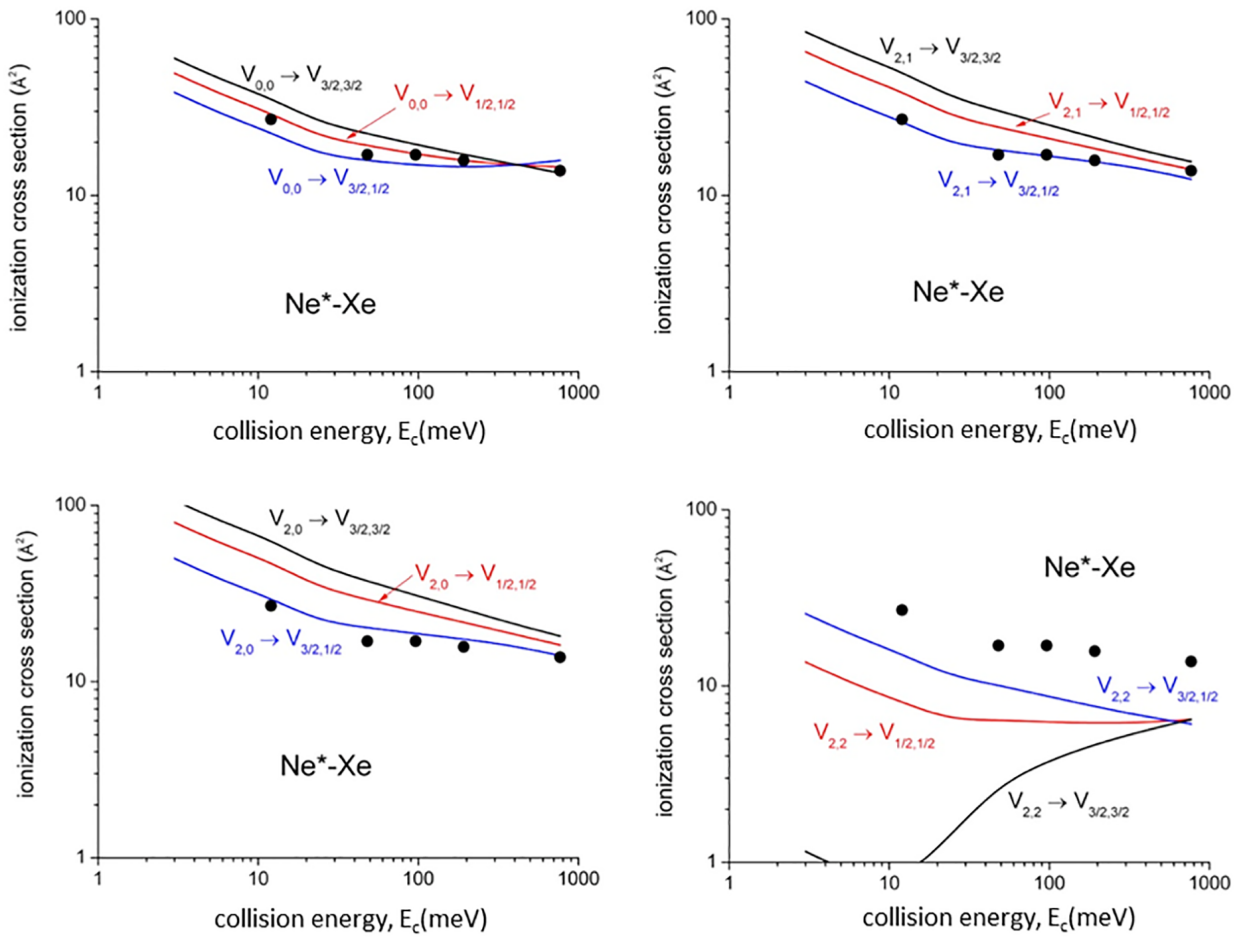
State-to-state total ionization cross section for Ne*–Xe as a function of *E*_c_. The comparison with early experimental results (black points, data from ref (42)) refers to state averaged conditions and emphasizes differences with respect to state-to-state results, while their statistical average is consistent with the experimental determination.

**Figure 6:**
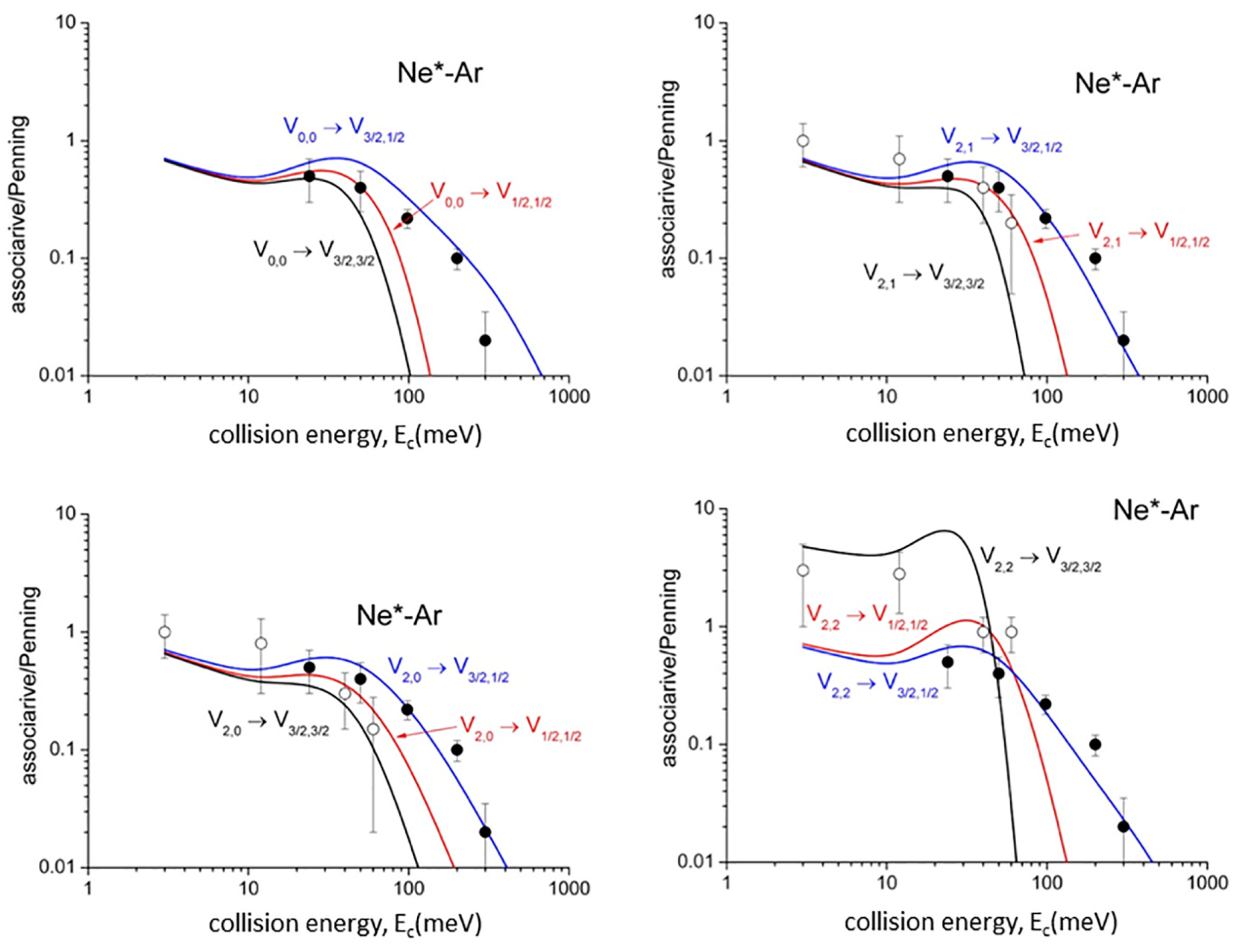
State-to-state associative/Penning ratios predicted for Ne*–Ar. The comparison involves experimental results (black points, data from ref (51)) referred to state averaged conditions. Recent data, measured with Ne*(^3^P_2_) beams state-selected in Ω = 2, 1, 0 quantum states, are also reported (open circles) for a further comparison (data from refs (43−45)).

**Figure 7:**
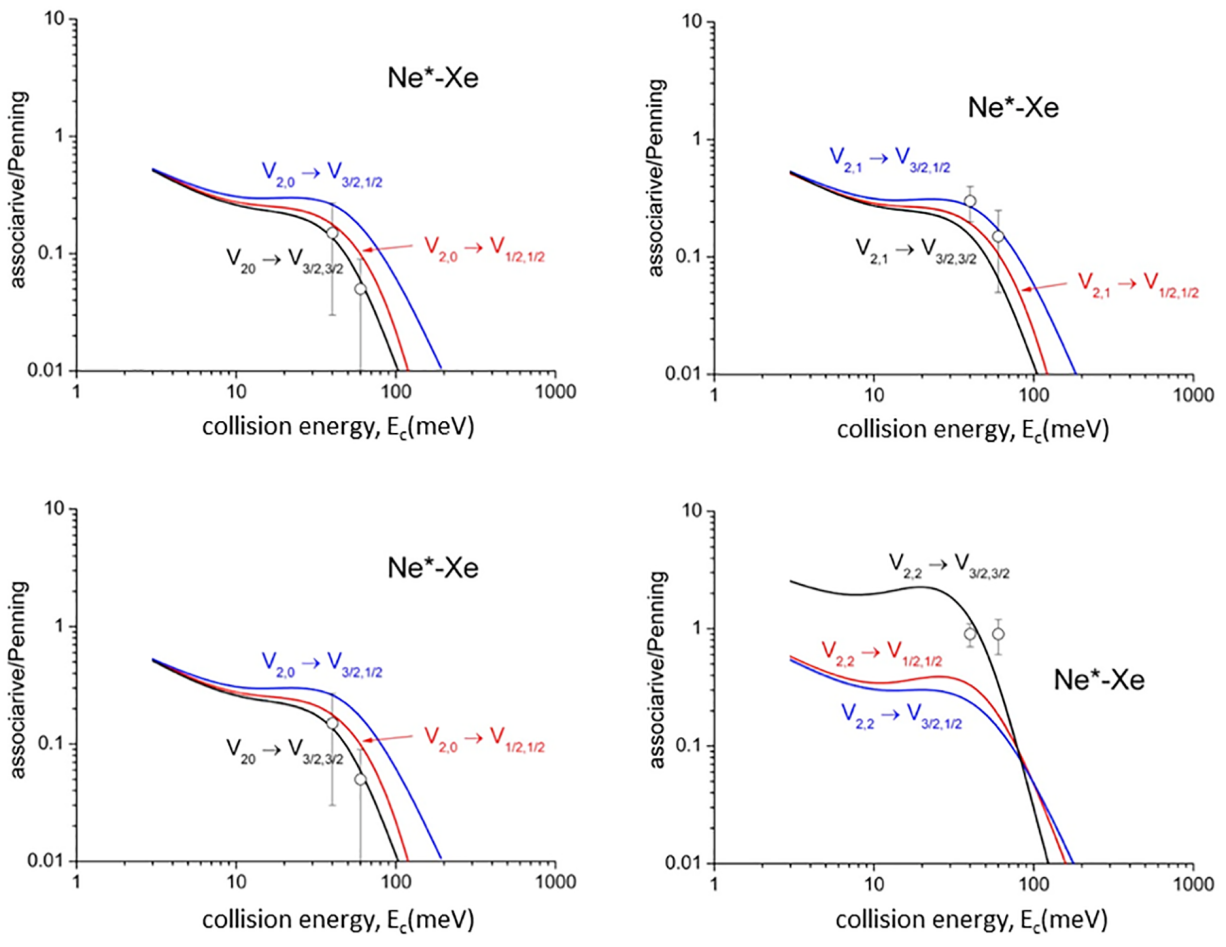
State-to-state associative/Penning ratios predicted for Ne*–Xe. The comparison includes only results of recent experiments (open circles). Data from ref (45).

Present optical potential formulations have been exploited to calculate, within a semiclassical method,^21,22,26,27^ state-to-state ionization cross sections over a wide *E*_c_ range. Such calculations directly provide also the product BRs, defining the relative probability of selected channels.^3^Figures 4, 5, 6, and 7 compare theoretical predictions with experimental data from several laboratories including our own. All experimental data have been obtained in high-resolution molecular beam experiments: our apparatus has been illustrated in previous papers^31,35^ and SI. Therefore, this treatment attempts to give, for the first time and for all Ne*–Ng systems, an internally consistent rationalization of most relevant experimental findings^1,3,35,42−45,51^

Pronounced differences in state-to-state total ionization cross sections, which directly relate to the different strengths and radial dependences of Γ_|*J*Ω→*J*′Ω′⟩_ components, are obtained. Representative experimental results^42^ with non-state-selected reagents in a wide *E*_c_ range are reported in Figures 4 and 5 (black points). Good agreement between theoretical predictions and experimental data, both in their absolute values and in *E*_c_ dependences, is obtained for values of the cross sections averaged over the statistical distribution of quantum states accessible in the experimental conditions.^42^

Cross section ratios, , representing the relative formation probability of the ionic aggregate [Ng^+^–Ne] (*associative ion*) with respect to the Ng^+^ (*Penning ion*), are also determined. The state-to-state BRs for associative to Penning ionization, , as a function of *E*_c_, are plotted in Figure 6 with non-state-selected experimental data (black points) from our laboratory.^51^ The experimental data compare well with the statistical average of the present calculations. However, the most important comparison is performed in Figures 6 and 7 with data by the Osterwalder group^43−45^ (open circles) recorded using a state-selected Ne* beam in *J* = 2 and Ω = 2, 1, 0 sublevels, indicating a good agreement with the state-to-state selectivity predicted here.

### Penning Ionization Electron Spectra

Ne*–Kr PIES experiments^1−3^ permitted us to separate contributions of different entrance and exit channels, referred to specific *J* levels of the Ne*(^3^P_2,0_) reagent and the Kr^+^(^2^P_3/2,1/2_) product. The large SO splitting in Kr^+^ and Xe^+^ allowed individual contributions to be resolved in the PIES data, measured as a function of *E*_c_ for both Ne*–Kr and Ne*–Xe systems.^1^ Similar experiments with Ne*–Ar have not been done, since SO splittings in Ne* reagent (0.097 eV) and in Ar^+^ product (0.177 eV) are comparable, making it difficult to separate the contribution of different SO states in entrance and exit channels.

### Dependence of the Observables on Optical Potential Features

**Figure 8:**
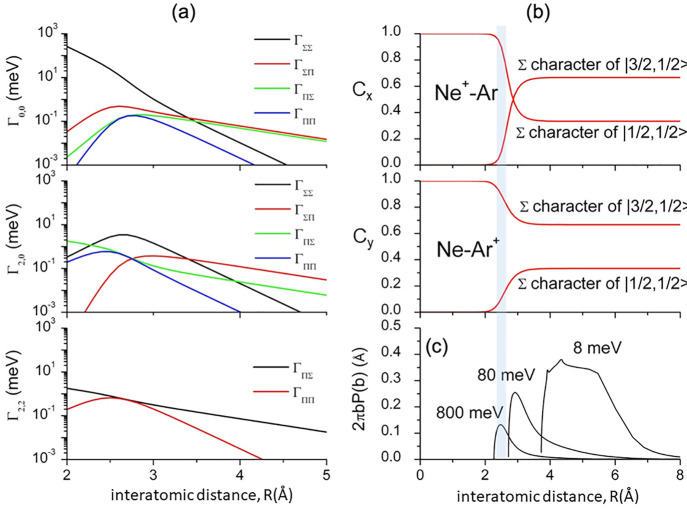
(a) Individual contributions to Γ, associated with different molecular symmetries Λ and Λ′ of entrance and exit channels of Ne* + Ar, plotted as a function of *R*. (b) Some details of the (0,0 −3/2,1/2) channel: the Σ character degree in entrance (*C*_*x*_) and exit (*C*_*y*_) channels (Figure 2) are plotted as a function of *R*. (c) Reaction probability *P*(*b*) at each impact parameter *b* and distance intervals probed at three selected *E*_c_ (thermal–hyperthermal range). For *E*_c_ < 3–4 meV (subthermal conditions, *T* ≤ 50 K) mainly probed distances are significantly larger than 4 Å, where *C*_*x*_ and *C*_*y*_ are weakly perturbed and tend to assume asymptotic-statistical values. Vertical gray area confines the turning points region for reactive oxidation collisions.

The proposed methodology clearly indicates how the various experimental findings depend on basic features of real and imaginary parts of the optical potential and how their role is modulated by *E*_c_ and the selected channel. While *V*_t_ controls the dynamics of reagent approach and product removal defining the *R* region mainly probed by the system at each *E*_c_, Γ determines the reaction probability for each assumed configuration of the TS in the probed *R* region. Important selectivity in the reaction dynamics emerges by deconvoluting from each state-to-state Γ component the contributions assigned to each Λ and Λ′ quantum number pair. These pairs describe the molecular symmetry of the system before and after the electron exchange. The channel |2,2⟩ →|3/2,3/2⟩ always shows the smallest cross sections that tend to vanish at low *E*_c_. This behavior can be rationalized by noting that this channel is exclusively governed by *A*_Π–Π_, a very weak coupling term effected by an electron exchange between valence orbitals aligned perpendicularly to *R* and the overlap of which is small and rapidly vanishing with *R*. For all other channels, the observed behavior arises from a combined effect of Σ and Π molecular character in the interaction driving the collision. The Ne*–Ar system has been considered as representative of the complete phenomenology, and the analysis has been focused on three different entrance channels, namely, |0,0⟩, |2,0⟩, and |2,2⟩, and on the same exit channel, |3/2,1/2⟩. Note that the |2,1⟩ channel behaves similarly to |2,0⟩. Deconvoluted results, obtained as a function of *R*, are plotted in Figure 8a. We emphasize that small and large distances identify, respectively, *R* regions mainly probed by experiments at high and low *E*_c_, respectively. From the figure, it appears that for the |0,0⟩ entrance channel the direct mechanism of Σ–Σ type is dominant at short distance, where the system assumes full Σ molecular character in both the initial and final states. In this selected quantum configuration, the Ne*–Ar chemi-ionization is dominated by the electron exchange inside the molecular [(Ne···Ng)^+^]^*e*−^ TS, promoting a chemical (oxidation) phenomenon. For |2,0⟩, the same mechanism is prevalent only at intermediate *R*, where the transition from atomic to molecular states is emerging and *C*_*x*_ and *C*_*y*_ associated with the Ne^+^ reagent ionic core and Ar^+^ product change quickly, as shown in Figure 8b, since SO couplings are destroyed in the electric field associated with the anisotropic interaction potential.

For all channels considered here, including the |2,2⟩ entrance one, the Π–Π direct (exchange-oxidation) mechanism plays a minor role at short *R*, since the exit channel tends to assume pure Σ symmetry. Contrarily, in all cases indirect Σ–Π and Π–Σ mechanisms become dominant for *R* ≥ 3.5 Å, where small changes in *C*_*x*_ and *C*_*y*_, induced by weak interactions, tend to reduce the validity of the optical selection rules stimulating radiative effects.^41,42^

By exploitation of semiclassical cross section calculations, it is possible to characterize the *R* regions mainly probed at each *E*_c_. Figure 8b,c reports this information for the (0,0 −3/2,1/2) channel and clearly confirms that, under hyperthermal conditions, the direct mechanism (chemical-oxidation) is dominant (lower *R*), while under thermal conditions direct and indirect mechanisms become competitive (intermediate *R*). Under subthermal conditions, where only a full quantum mechanical cross section calculation is appropriate, only the indirect mechanism (radiative-physical) is effective (larger *R*), being driven exclusively by weak isotropic long-range interactions. Figure 8b,c also shows the turning point region where oxidation collisions show the greatest reactivity.

Experimental findings related to  ratios (Figures 6 and 7) probe other details of state-to-state components of the optical potential. In particular, the highest  value for the |2,2⟩ entrance channel at low and intermediate *E*_c_ arises from the softer repulsive wall of *V*_|2,2⟩_, emerging at intermediate *R* highlighted in Figure 1c,d, which allows a more prominent approach of reactants that favors the trapping in the potential well of the exit channels. This observation represents a stereodynamical feature clearly evident in experiments of the Osterwalder group.^43−45^ At high *E*_c_, the associative/Penning ionization ratio, , falls off fast, as experimentally observed,^51^ since the reactions provide ionic products confined in the repulsive wall of the exit channels, which lead to dissociated ions. At very low *E*_c_,  ratios are affected by the weak long-range attraction, where the anisotropic nature of the Ne^+^ core is shielded by the isotropic behavior of the excited 3s electron.

**Figure 9:**
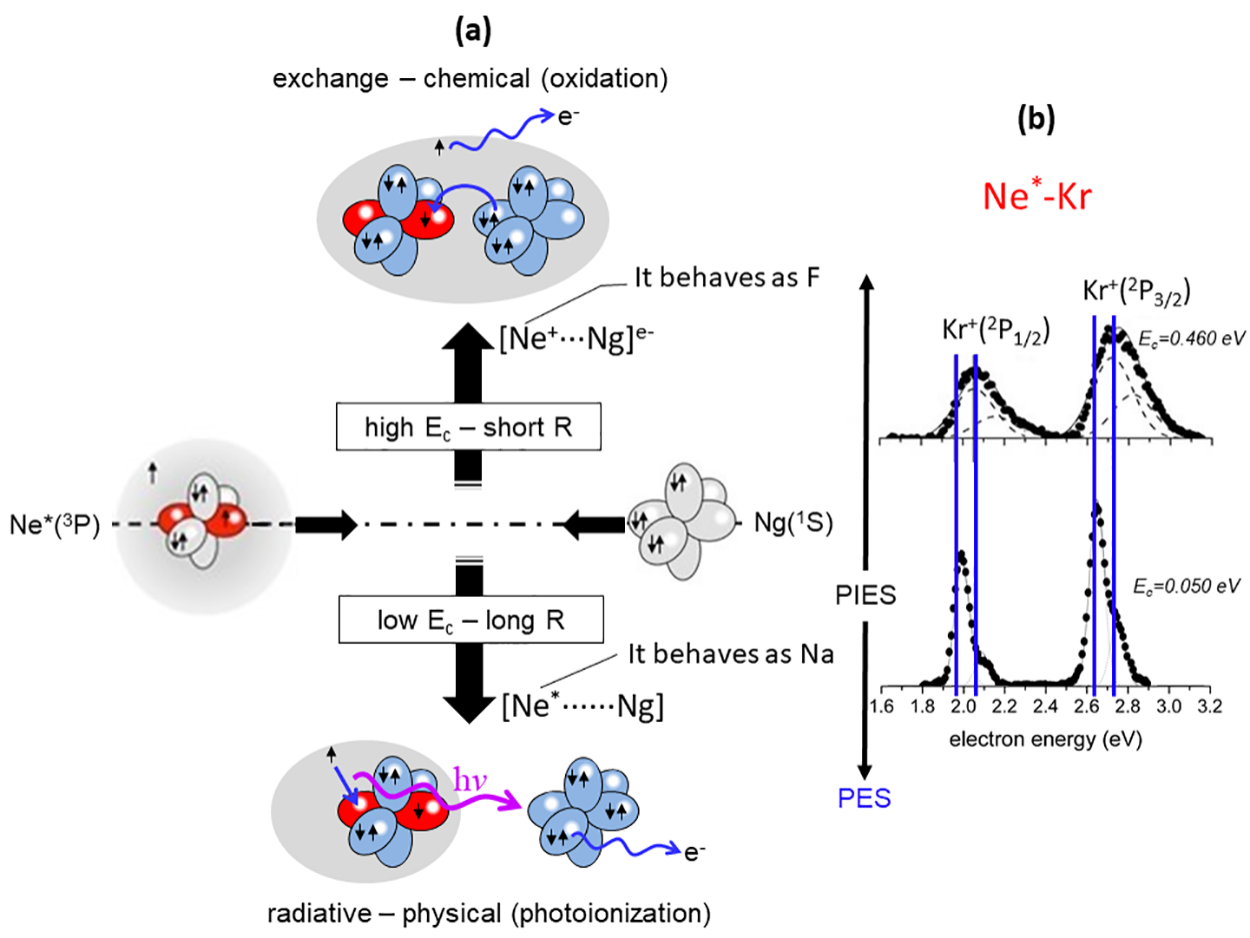
(a) Schematic view of two mechanisms in chemi-ionizations. (b) Ne*–Kr PIESs where vertical blue lines indicate peak positions from Ne(I) photoionization spectrum (PES): at higher *E*_c_ electron spectra are very different from PES indicating a chemical interaction inside the formed [(Ne···Ng)^+^]^*e*−^ TS (oxidation mechanism), while at very low *E*_c_, they become very similar to PES since the [Ne*······Kr] TS evolves via a photoionization process.

Finally, the most important evidence of reaction mechanism modulation is provided by the *E*_c_ dependence of measured PIES, resolved for *J* levels of entrance and exit channels. Specifically, very low *E*_c_ leads to the exclusive formation of diatomic adducts [Ne*······Ng] (eq 2 and Figure 9a), binding by weak noncovalent interactions, and the observables are consistent with those of pure photoionization spectra (PES) generated by a radiative (physical) phenomenon (Figure 9b). At high *E*_c_, the appearance of chemical interaction components modifies the TS in the molecular [(Ne···Ng)^+^]^*e*−^ structure (Figure 9a), where Ne^+^–Ng and Ne–Ng^+^ configurations couple by CT. Consequently, significant changes in peak shape and position appear in measured PIES with respect to PES (Figure 9b), indicating the emergence of a chemical-oxidation reaction. Figure 9 emphasizes these changes for Ne*–Kr pointing out the main characteristics of the two mechanisms discussed for chemi-ionization reactions (for Ne*–Xe, see details in SI).

## Conclusions

This new treatment provides unique information on the stereodynamics of chemi-ionization reactions, which are relevant in flames, astrochemistry, plasmas, and nuclear fusion.^1−3,53−56^ Electronic angular momentum couplings and orbital alignment are properly accounted for to describe the selectivity of each state-to-state channel. Since collision complexes are rotating adducts, their features must be consistent with angular momentum couplings confined in specific Hund’s cases.^52^ The emergence of the direct (exchange-oxidation) mechanism corresponds to the passage from Hund’s case c to Hund’s case a, while the indirect (radiative) mechanism operates when the transition concerns Hund’s e to Hund’s c cases.

The proposed methodology (i) identifies two important markers (C_*x*_ and C_*y*_), which permit a description of adiabatic and nonadiabatic effects through the use of simple phenomenological equations, (ii) includes previous theoretical descriptions since 1970,^27,41,42^ (iii) is a simple and general treatment reproducing experimental results from our and other laboratories since 1981,^1,3,42−45,51,53,54^ (iv) clarifies that exchange and radiative mechanisms are not alternative but simultaneously operative with a relative weight that changes with *E*_c_ and depends on the investigated state-to-state channel, and (v) clarifies for the first time that chemi-ionizations are prototype gas phase elementary oxidation processes that can be probed by PIES, a spectroscopy of TS not allowed in the condensed phase.

## Supporting Information Available

Potential energy formulation, the definition of state-to-state Γ_|*J*Ω→*J*′Ω′⟩_ components, and the PIES of Ne*–Xe (PDF)
