# Supplementary material for: Quantum-State Controlled Reaction Channels in Chemi-ionization Processes: Radiative (Optical–Physical) and Exchange (Oxidative–Chemical) Mechanisms
Source: Acc Chem Res. 2020 Sep 15;53(10):2248–60. doi: 10.1021/acs.accounts.0c00371 (PMC8011800; doi:10.1021/acs.accounts.0c00371)
Supplement: Supplementary file 1 — ar0c00371_si_001.pdf [file ar0c00371_si_001.pdf]

SUPPORTING INFORMATION to the paper

Quantum-State Controlled Reaction Channels in Chemi-ionization Processes: Radiative (Optical−Physical) and Exchange (Oxidative−Chemical) Mechanisms

Stefano Falcinelli[a,*], James M. Farrar[b], Franco Vecchiocattivi[a], and Fernando Pirani[c,d]

[a]*Dipartimento di Ingegneria Civile ed Ambientale, Università di Perugia, 06125 Perugia, Italy*

[b]*Department of Chemistry, University of Rochester, Rochester NY 14627, USA*

[c] *Dipartimento di Chimica, Biologia e Biotecnologie, Università di Perugia, 06123 Perugia, Italy*

[d]*Istituto di Scienze e Tecnologie Chimiche "G. Natta" CNR-SCITEC, 06123 Perugia, Italy.*

The potential energy formulation

The adopted formulation of the real component V_t of the optical potential, providing in the entrance channels its dependence on the intermolecular distance R, leads to this expression:[1,2]

$$V_t(R) = S(R)\, V(R)^{neut.-neut.} + \left(1 - S(R)\right) V(R)^{ion-neut.}$$

where $V(R)^{neut.-neut.}$ and $V(\mathrm{R})^{\mathrm{ion-neut.}}$ have been represented by the Improved Lennard Jones function, whose general form is [3]

$$V_{ILJ}(R) = \varepsilon \left[\frac{m}{n(R) - m} \left(\frac{R_m}{R}\right)^{n(R)} - \frac{n(R)}{n(R) - m} \left(\frac{R_m}{R}\right)^{m} \right]$$

with

$$n(R) = \beta + 4\left(\frac{R}{R_m}\right)^2$$

Here, ε is the potential well depth and R_m is its location, while $n(R)$ defines the hardness of the repulsive wall and the radial modulation of the attraction. The switching function $S(R)$, that accounts

for the transition from the neutral-neutral to the ion-neutral representation of the interaction, as previously [1] it has been defined as

$$S(R) = \frac{1}{1 + e^{\left(\frac{R_o - R}{d}\right)}}$$

Here R_o is the distance where the two combined limiting potential forms have the same weight, while d describes how fast the transition occurs. However, for the complete representation of V_t it is necessary to take into account additional aspects:

a) At large *R*, where the neutral-neutral representation is dominant and the system exhibits a substantial isotropic behavior typical of an alkaline atom interacting with a noble gas partner, the ILJ formulation of the interaction appears to be complete.

b) At intermediate and short *R*, where the anisotropic role of the ionic core of the metastable atom is emerging, it is necessary to take into account for contributions due to its open shell "P" nature.[4]

c) Also in the exit channels the open shell nature of the atomic ion in "P" state controls the involved interaction anisotropy

Therefore, following the guidelines extensively developed in our laboratory,[4-6] it is possible to describe the interaction energy, when an open "P" shell atom or ion approaches an closed shell species, by effective adiabatic potential energy curves, that include the contributions associated to pure Σ and Π molecular states, defined by the electronic quantum number Λ=0 and Λ=1 and identified as $V_\Sigma$and V_Π, mixed by spin orbit effects. It is proper to employ for such a description a weighted sum of V_0 and V_2 Legendre-expansion radial coefficients, defined as $V_0 = \frac{1}{3}(V_\Sigma + 2V_\Pi)$ and $V_2 = \frac{5}{3}(V_\Sigma - V_\Pi)$. The inverse formulas are simply given by $V_\Sigma = V_0 + \frac{2}{5}V_2$ and. $V_\Pi = V_0 - \frac{1}{5}V_2$ In this way, while V_0 describes the spherical average interaction component, all anisotropic contributions, arising from quantized spatial orientations of the valence orbitals of the open shell atom or ion within the interacting complex, are directly taken into account through the use of the anisotropic term V_2 . In the case of ^3P_J (or ^2P_J) open shell atomic species, like Ne^* (or Ne^+) and Kr^+, characterized

by a reversed sequence of spin orbit sublevels (see Figure S1), le effective adiabatic potential energy curves $V_{|J,\Omega>}$ (Ω is the quantum number defining the absolute projection of **J** along R) have been defined for all channels and formulated as:

Entrance channels

$$V_{|0,0>} = V_0 + \frac{1}{10} V_2 + \frac{1}{2}\Delta_0 + \frac{1}{2}\left(\frac{9}{25} V_2^2 + \Delta_0^2 - \frac{2}{5} V_2 \Delta_0\right)^{1/2}$$

$$V_{|2,0>} = V_0 + \frac{1}{10} V_2 + \frac{1}{2}\Delta_0 - \frac{1}{2}\left(\frac{9}{25} V_2^2 + \Delta_0^2 - \frac{2}{5} V_2 \Delta_0\right)^{1/2}$$

$$V_{|2,1>} = V_0 + \frac{1}{10} V_2 + \frac{1}{2}\Delta_1 - \frac{1}{2}\left(\frac{9}{25} V_2^2 + \Delta_1^2\right)^{1/2}$$

$$V_{|2,2>} = V_0 - \frac{1}{5} V_2$$

Exit channels

$$V_{|1/2,1/2>} = V_0 + \frac{1}{10} V_2 + \frac{1}{2}\Delta + \frac{1}{2}\left(\frac{9}{25} V_2^2 + \Delta^2 - \frac{2}{5} V_2 \Delta\right)^{1/2}$$

$$V_{|3/2,1/2>} = V_0 + \frac{1}{10} V_2 + \frac{1}{2}\Delta - \frac{1}{2}\left(\frac{9}{25} \mathrm{V}_2^2 + \Delta^2 - \frac{2}{5} \mathrm{V}_2 \Delta\right)^{1/2}$$

$$V_{|3/2,3/2>} = V_0 - \frac{1}{5} V_2$$

where Δ_0, Δ_1 and Δ are the energy splittings between fine atomic sublevels, whose definition and values are given in Figure S1. Major details on ^3P_J interactions are given in ref. 7.

As indicated above, in the entrance channels the V_0 term, coinciding with the isotropic component of $V_t(R)$, exhibits a mixed nature, accounting for the gradual passage from neutral-neutral to ion-neutral system (surrounded by an electron in a Rydberg state), as R decreases. In the exit channels, a pure ion- neutral system operates. In the two cases, the V_0 terms have been defined as combination or simple ILJ functions, respectively. Moreover, for both entrance and exit channels, V_2, that accounts for all anisotropic interaction contributions, has been represented by an exponential decreasing function, defined by a pre-exponential factor A and an exponent α, plus an additional

contribution $\frac{C_a}{R^6}$. For entrance and exit channels the modulus of the exponential function is the same, while its sign is negative for exit and positive for entrance, since related to *bonding* and *antibonding* stabilization effects by charge transfer that arise from the configuration interaction between entrance and exit channels of the same symmetry,[4,7] as depicted in Figure 1 of the main text. The additional contribution accounts for the role of polarizabilty anisotropy on asymptotic behavior of V_2 . All the potential parameters are given in Table S1. They have been obtained semi-empirically following the guidelines described in refs 4,6,7 that have been suggested by the phenomenological investigation of several systems involving open shell atoms and ions.

Since the sign the exponential contribution to V_2 is positive for entrance and negative for exit channels, this interaction potential formulation leads to a different correlation between atomic states, representative of the system at long range separation distances, where $|V_2| \ll \Delta_i$, and molecular states of the same system emerging at short range, where $|V_2| \gg \Delta_i$ (See Figure 1 of the main text and Figure S2). Moreover, it has been also deduced that the Σ and Π character of involved potential energy curves $V_{|J,\Omega>}$ at all R values can be evaluated from the following relations:[5]

$$V_{|2,0>}, V_{|2,1>}, V_{|3/2,1/2>} = cos^2\alpha\, V_\Sigma + sin^2\alpha\, V_\Pi$$

$$V_{|0,0>}, V_{|1/2,1/2>} = sin^2\alpha\, V_\Sigma + cos^2\alpha\, V_\Pi$$

where

$$\cos^2\alpha = \frac{1}{2} + \frac{\left(1 - \frac{9V_2}{5\Delta}\right)}{4\sqrt{2}\sqrt{1 + \left[\left(\frac{1 - \frac{9V_2}{5\Delta}}{2\sqrt{2}}\right)\right]^2}}$$

Again, in all cases only the exponential contribution to the V_2 component with its proper sign must be taken into the previous equation. This boundary arises from the meaning of the exponential term which is the only one selectively representing the configuration interaction anisotropy by charge transfer, which is the exclusive component determining the formation of molecular states. These formulas agree with the following asymptotic conditions (See Figure S2): at short distances, all

potential energy curves must represent states having a pure Σ or Π character, while at large distances, where spin orbit coupling is dominant, a mixing of the characters occurs (see Figure 2 of the main text). Note also that the different behavior of Ne *-Ng (or Ne^+-Ng), with respect to Ne-Ng^+, arises from the opposite sign of V_2 component and from the different role of the spin orbit mixing, defined by the different values of Δ_0, Δ_1 and Δ. The behavior of $V_{|2,2>}$ and $V_{|\frac{3}{2},\frac{3}{2}>}$ curves, effective in the entrance and exit channels, respectively, is not discussed in detail because they show at all distances a pure Π character. Additional details on the adiabatic correlation between atomic and molecular states, both in entrance and exit channels, defined in terms of proper quantum number, are given in Figure S2, where also the meaning of C_x and C_y coefficients, with their relevance in the formulation of state to state Γ (See the main text and below) is justified. Such coefficients must be considered as the proper markers of the system evolution along each reaction channel.

It has been also demonstrated [4,6,7] that *non adiabatic* effects, promoted by changes in the electronic angular coupling schemes, describing the transition from atom-atom to molecular states, manifest with the highest probability at a distance where $|V_2|$ is comparable with Δ_i.

The definition of state-to state $\Gamma_{|J,\Omega \to J',\Omega'>}$ components

The important new aspect is that here we are able to evaluate the relative role of two mechanisms for each state to state channel with its dependence on the collision energy or on the distance range of R mainly probed. In particular, simple-operative exponential functions have been adopted for $A_{\Sigma-\Sigma}$, $A_{\Pi-\Pi}$, $A_{\Sigma-\Pi}$ and $A_{\Pi-\Sigma}$ coupling terms whose parameters are given in Table S2.

The first two terms represent a defined fraction of V_2, while the last two have been obtained adopting criteria given in refs 8,9. It is important to remark that overlap effects with the continuum wave function of emitted electrons (see the main text) are here indirectly enclosed in the pre-exponential factor and this allows to better explicit the couplings between discrete quantum states which are more effective for the electronic rearrangements within the collision complex.

On the grounds of the main points of the proposed methodology discussed in the text , and considering the correlation diagram between atomic and molecular states reported in Figure S2, we have obtained explicit relations for state-to state $\Gamma_{|J,\Omega\rightarrow J',\Omega'>}$ components of the optical potential (see eq. (1) of the main text), represented as weighted averages of $A_{\Lambda-\Lambda'}$ couplings, where the relative weights (their sum is normalized to 1 in each channel) are just given in terms of C_x and C_y coefficients:

$$\Gamma_{|0,0\rightarrow\frac{1}{2},\frac{1}{2}>} = A_{\Sigma-\Sigma}C_x(1-C_y) + A_{\Sigma-\Pi}\, C_x\, C_y + A_{\Pi-\Sigma}\,(1-C_x)(1-C_y) + A_{\Pi-\Pi}\,(1-C_x)C_y\ ;$$

$$\Gamma_{|2,0\rightarrow\frac{1}{2},\frac{1}{2}>} = A_{\Sigma-\Sigma}(1-C_x)(1-C_y) + A_{\Sigma-\Pi}\,(1-C_x)C_y + A_{\Pi-\Sigma}\,C_x(1-C_y) + A_{\Pi-\Pi}\,C_xC_y\ ;$$

$$\Gamma_{|2,1\rightarrow\frac{1}{2},\frac{1}{2}>} = A_{\Sigma-\Sigma}\,\tfrac{3}{4}\,(1-C_x)(1-C_y) + A_{\Sigma-\Pi}\,\tfrac{3}{4}\,(1-C_x)C_y + A_{\Pi-\Sigma}\,\left(\tfrac{3}{4}C_x+\tfrac{1}{4}\right)(1-C_y) + A_{\Pi-\Pi}\,\left(\tfrac{3}{4}C_x+\tfrac{1}{4}\right)C_y\ ;$$

$$\Gamma_{|2,2\rightarrow\frac{1}{2},\frac{1}{2}>} = A_{\Pi-\Sigma}\,(1-C_y) + A_{\Pi-\Pi}\,C_y\ ;$$

$$\Gamma_{|0,0\rightarrow\frac{3}{2},\frac{1}{2}>} = A_{\Sigma-\Sigma}C_xC_y + A_{\Sigma-\Pi}\,C_x\,(1-C_y) + A_{\Pi-\Sigma}\,(1-C_x)C_y + A_{\Pi-\Pi}\,(1-C_x)(1-C_y)\ ;$$

$$\Gamma_{|2,0\rightarrow\frac{3}{2},\frac{1}{2}>} = A_{\Sigma-\Sigma}(1-C_x)C_y + A_{\Sigma-\Pi}\,(1-C_x)(1-C_y) + A_{\Pi-\Sigma}\,C_xC_y + A_{\Pi-\Pi}\,C_x(1-C_y)\ ;$$

$$\Gamma_{|2,1\rightarrow\frac{3}{2},\frac{1}{2}>} = A_{\Sigma-\Sigma}\,\tfrac{3}{4}\,(1-C_x)C_y + A_{\Sigma-\Pi}\,\tfrac{3}{4}(1-C_x)(1-C_y) + A_{\Pi-\Sigma}\,\left(\tfrac{3}{4}C_x+\tfrac{1}{4}\right)C_y + A_{\Pi-\Pi}\,\left(\tfrac{3}{4}C_x+\tfrac{1}{4}\right)(1-C_y)\ ;$$

$$\Gamma_{|2,2\rightarrow\frac{3}{2},\frac{1}{2}>} = A_{\Pi-\Sigma}\,C_y + A_{\Pi-\Pi}\,(1-C_y)\ ;$$

$$\Gamma_{|0,0\rightarrow\frac{3}{2},\frac{3}{2}>} = A_{\Sigma-\Pi}\,C_x + A_{\Pi-\Pi}\,(1-C_x)\ ;$$

$$\Gamma_{|2,0\rightarrow\frac{3}{2},\frac{3}{2}>} = A_{\Sigma-\Pi}\,(1-C_x) + A_{\Pi-\Pi}\,C_x\ ;$$

$$\Gamma_{|2,1\rightarrow\frac{3}{2},\frac{3}{2}>} = A_{\Sigma-\Pi}\,\tfrac{3}{4}(1-C_x) + A_{\Pi-\Pi}\,\left(\tfrac{3}{4}C_x+\tfrac{1}{4}\right)\ ;$$

$$\Gamma_{|2,2\rightarrow\frac{3}{2},\frac{3}{2}>} = A_{\Pi-\Pi}$$

The PIES of Ne^{*}-Xe

The importance of calculated *state-to-state cross sections* is discussed in the main text. Their ratios, averaged over the *Ω* quantum numbers in order to obtain the exclusive dependence on the *J* spin-orbit

sublevels of both neutral reactants and ionic products, allow a comparison with peak area ratios extracted from PIES's measured as a function of the collision energy. Results for Ne^*-Xe as a function of the collision energy are plotted in the left lower panel of Figure S3, where they are indicated as R_i ratios. They show that the processes involving metastable atoms in J=0 highlight their relative role at higher collision energies. As discussed in the main text, this effect arises from the increased value of the C_x coefficient at short R, related to an emerging Σ molecular character which promotes a more efficient ionization. Moreover, in the probed collision energy range, the ratios referred to final states with $J_f = \frac{1}{2}$ and $J_f = \frac{3}{2}$ remain almost constant, and they amount to about 0.5 since in both exit channels their degeneracy, averaged over permitted Ω (where $\Omega = \frac{1}{2}$ corresponds to $J_f = \frac{1}{2}$, while $\Omega = \frac{1}{2}, \frac{3}{2}$ to $J_f = \frac{3}{2}$), is respectively 1:2 and Σ and Π molecular characters maintain always a nearly statistical weight (i.e. 1:2). For two of the four ratios such a comparison appears to be only in semi-quantitative agreement with the experimental results, while for the other two is all right. However, considering the difficulties involved in the separation of individual spin orbit contributions from measured energy dependence of PIESs, also for such quantities the theoretical description appears to be consistent with the experimental observables.

References

1. Brunetti, B.G., et al. The Stereodynamics of Penning ionization of water by metastable neon atoms. *J. Chem. Phys.* **139**, 164305 (2013).
2. Falcinelli, S., et.al. The electron couplings in the transition states: the stereodynamics of state to state autoionization processes. *J. Chem. Phys.***150,** 044305 (2019).
3. Pirani, F., et al. Beyond the Lennard Jones model: a simple and accurate potential function probed by high resolution scattering data useful for molecular dynamics simulations. *Phys. Chem. Chem. Phys.***10**, 5489-5503 (2008).
4. Pirani, F., et al. Experimental benchmarks and phenomenology of interatomic forces: open-shell and electronic anisotropy effects. *Int. Rev. Phys. Chem.* **25**, 165-199 (2006).

5. Tosi, P., et al. The reaction of argon ions with hydrogen and deuterium molecules by crossed beams: Low energy resonances and role of vibronic levels of the intermediate complex. *J. Chem. Phys.* **99**, 985-1003 (1993).
6. Pirani, F., et al. Coupling by charge transfer: role in bond stabilization for open-shell systems and ionic molecules and in harpooning and proton attachment processes. Mol. Phys. **98**, 1749-1762 (2000).
7. Aquilanti, V., et al. Molecular beam studies of weak interactions for open-shell systems: The ground and lowest excited states of rare gas oxides. *J. Chem. Phys.* **89**, 6157-6164 (1988).
8. Falcinelli, S., Vecchiocattivi, F., Pirani, F. Adiabatic and Nonadiabatic Effects in the Transition States of State to State Autoionization Processes. *Phys. Rev. Lett.* **121**, 163403 (2018).
9. 28. Falcinelli, S., Vecchiocattivi, F., Pirani, F. The electron couplings in the transition states: the stereodynamics of state to state autoionization processes. *J. Chem. Phys.* **150**, 044305 (2019).

Table S1

Potential parameters of V_t

		Ne^*-Ar	Ne^+-Ar	Ne-Ar^+	Ne^*-Xe	Ne^+-Xe	Ne-Xe^+
Isotropic component	ε *(meV)*	6.08	167.1	41.55	13.8	268.6	29.8
	R_m *(Å)*	5.17	2.73	2.84	5.20	3.02	3.23
	β	6.85	8.0	9.0	6.50	8.0	8.5
	R_0 *(Å)*	3.85			3.85		
	d (Å)	0.55			0.55		
Anisotropic component	*A (meV)*		1.233×10^7			1.907×10^7	
	α *(Å^{-1})*		4.32			4.32	
	C_2 *(meV Å^6)*			1200.			2550.

Table S2

Strength and radial dependence of $A_{\Sigma\Sigma}$, $A_{\Pi\Pi}$, $A_{\Sigma\Pi}$, and $A_{\Pi\Sigma}$ coupling terms provided by an exponential function of general form $A_i=B_i \exp(-\beta_i R)$. The exponent β_i is assumed to be 4.32 and 1.40 Å^{-1} for $A_{\Sigma\Sigma}$-$A_{\Pi\Pi}$ and $A_{\Sigma\Pi}$-$A_{\Pi\Sigma}$ *coupling* terms, respectively. The values of B_i (meV) are listed below. Those of Ne^*-Kr are also enclosed for a useful comparison

	$A_{\Sigma\Sigma}$	$A_{\Pi\Pi}$	$A_{\Sigma\Pi}$	$A_{\Pi\Sigma}$
Ne^*-Ar	1.48×10^6	2.96×10^5	148.	29.6
Ne^*-Kr	2.01×10^6	4.02×10^5	201.	40.2
Ne^*-Xe	2.29×10^6	4.58×10^5	229.	45.8

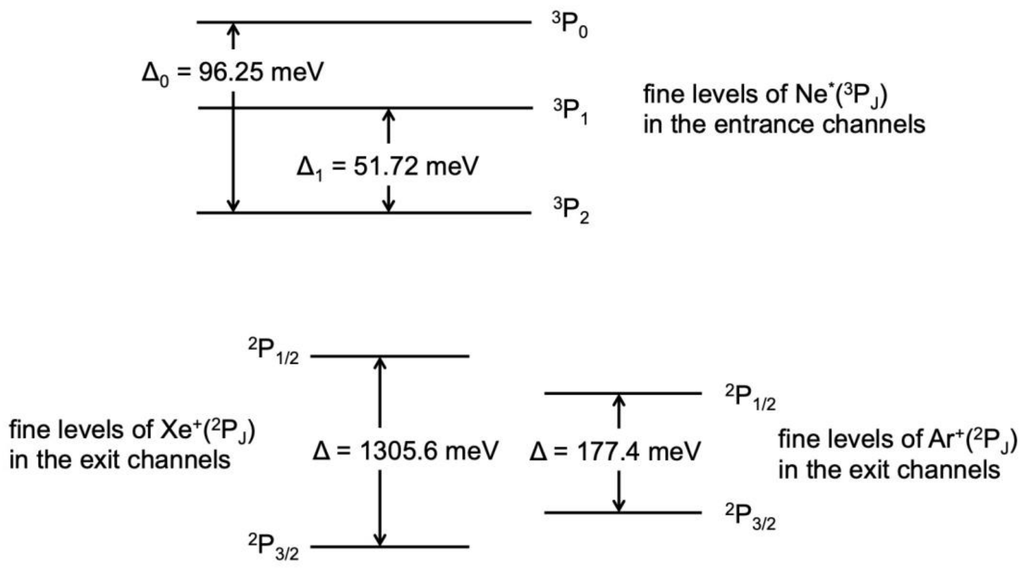

Figure S1 – The sequence of fine levels in entrance and exit channels. Note that, although $Ne^*(^3P_1)$ is not metastable and then it does not participate to the autoionization reactions, the Δ_1 splitting is involved in the representation of the effective adiabatic potentials given in this supporting information. Note that Δ_0 define also the SO splitting in $^2P_{1/2}$ and $^2P_{3/2}$ Ne^+ ion.

CORRELATION DIAGRAM BETWEEN ATOMIC AN MOLECULAR STATES

<table>
<tr><th colspan="5">ENTRANCE CHANNELS</th><th colspan="4">EXIT CHANNELS</th></tr>
<tr><th>Large R</th><th>Intermediate R</th><th>Short R</th><th>Σ character</th><th>Π character</th><th>Large-intermediate R</th><th>Short R</th><th>Σ character</th><th>Π character</th></tr>
<tr><td>atom-atom |J,Ω> states</td><td>ion-atom |J,Ω> states</td><td>molecular ion $^{2s+1}\Lambda_\Omega$ states</td><td></td><td></td><td>ion-atom |J,Ω> states</td><td>molecular ion $^{2s+1}\Lambda_\Omega$ states</td><td></td><td></td></tr>
<tr><td>|0,0></td><td>|1/2,1/2></td><td>$^2\Sigma_{1/2}$</td><td>C_x</td><td>$1-C_x$</td><td>|1/2,1/2></td><td>$^2\Pi_{1/2}$</td><td>$1-C_y$</td><td>C_y</td></tr>
<tr><td>|2,0></td><td>|3/2,1/2></td><td>$^2\Pi_{1/2}$</td><td>$2/5\ (1-C_x)$</td><td>$2/5\ C_x$</td><td>|3/2,3/2></td><td>$^2\Pi_{3/2}$</td><td>-</td><td>1</td></tr>
<tr><td>|2,1></td><td></td><td></td><td>$3/5\ (1-C_x)$</td><td>$3/5\ C_x + 1/5$</td><td></td><td></td><td></td><td></td></tr>
<tr><td>|2,2></td><td>|3/2,3/2></td><td>$^2\Pi_{3/2}$</td><td>-</td><td>4/5</td><td>|3/2,1/2></td><td>$^2\Sigma_{1/2}$</td><td>C_y</td><td>$1-C_y$</td></tr>
</table>

Figure S2 – In entrance and exit channels the sequence of molecular states is opposite because the bonding and antibonding effects due to the configuration interaction.

Entrance channels features:
The structure of the ionic adduct, surrounded by the excited electron in a Rydberg state, is of relevance to determine crucial features of the system at intermediate and short R.
Entrance channels boundaries:
A large R, C_x=0.333 and the two fine states J=2,0 of Ne* exhibit Σ and Π character in the 1:2 statistical ratio. Moreover, the global weight (sum of both character degrees) of |2,0> state is one half of that of |2,1> and |2,2>, because of the different degeneracy. With the R decreasing, the |2,1> state assumes a Π molecular character faster than |2,0> since the spin orbit mixing is characterized by $\Delta_1 < \Delta_0$ (see Fig. S1).
Exit channels boundaries:
At large R, C_y=0.667 for the |3/2,1/2> state and also in this case fine both levels exhibit Σ and Π character mixed in the 1[illegible]tatistical ratio.

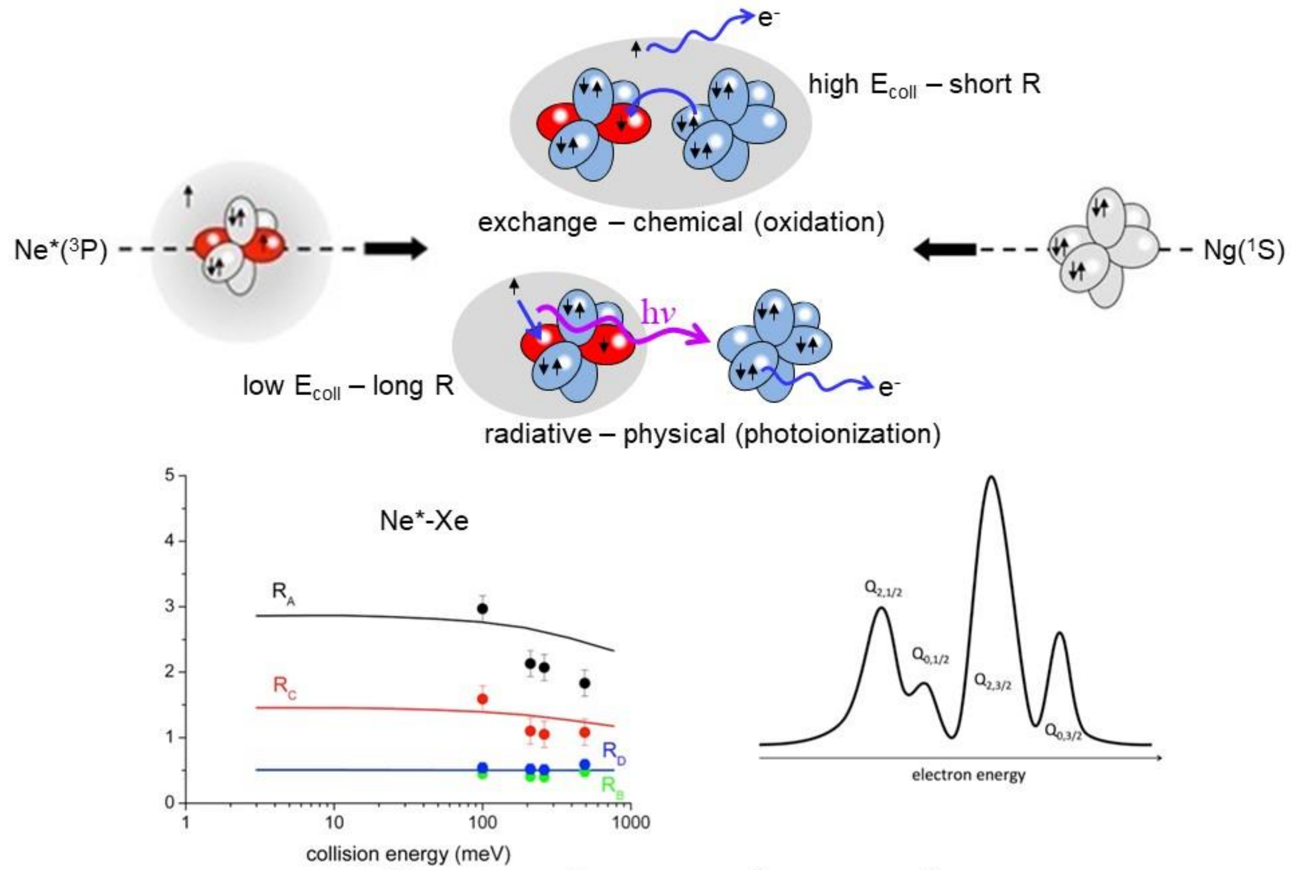

$$R_A = \frac{Q_{2,3/2}}{Q_{0,3/2}};\ R_B = \frac{Q_{0,1/2}}{Q_{0,3/2}};\ R_C = \frac{Q_{2,1/2}}{Q_{0,3/2}};\ R_D = \frac{Q_{2,1/2}}{Q_{2,3/2}};$$

FIGURE S3 - *Upper panel*: A schematic view of the two mechanisms of chemiionization processes with their main characteristics (see the main text). *Intermediate right panel:* qualitative relation between cross section of each spin orbit resolved channel and position and area of peaks in PIES's. *Intermediate left panel*: R_i values, representative of the cross section (or reaction probability) ratios of different channels $|J_i \to J_f\rangle$ respect to a reference one. Points, in the left panel, represent peak area ratios extracted from the analysis of PIES as depicted in the right panel. The continuous lines are the results of the present treatment carried out assuming a $^3P_2/^3P_0$ population ratio of about 3, as found for a Ne* beam generated by electron impact. At the bottom of the Figure is given the definition of R_i in terms of cross section ratios.[1]
